# A Self-Guided Web-Based Transdiagnostic Mental Health Program for People With Intellectual Disability: Single-Arm Trial

**DOI:** 10.2196/82246

**Published:** 2026-06-11

**Authors:** Peter Baldwin, Victoria Rasmussen, Wesley Grey, Julian Trollor, Jenna Zhao, Josephine Anderson, Chris Rule, Chloe Heck, Anita Gardner, Helen Christensen, Katherine Boydell

**Affiliations:** 1 Black Dog Institute, Hospital Road, Randwick, 2037, Australia, 61 405198608; 2 University of New South Wales Medicine and Health, Randwick, Australia; 3 National Centre of Excellence in Intellectual Disability Health, UNSW Medicine and Health, UNSW Sydney, NSW, Australia

**Keywords:** mental health, intellectual disability, digital, web-based, anxiety, depression

## Abstract

**Background:**

People with intellectual disability experience rates of mental illness up to 3 times higher than the general population, yet face significant barriers to care, including limited clinician expertise, diagnostic overshadowing, and exclusion from key mental health services. Electronic mental health interventions have demonstrated effectiveness in the general population and may address these barriers for people with intellectual disability by providing accessible, tailored treatment.

**Objective:**

This study examined the ability of Healthy Mind, a self-guided, web-based transdiagnostic mental health program designed specifically for people with borderline to mild intellectual disability, to reduce symptoms of anxiety and depression and improve functioning in people with intellectual disability.

**Methods:**

In a single-arm uncontrolled design, Australian residents aged ≥16 years with a diagnosis of borderline or mild intellectual disability, and mild-to-moderate symptoms of depression or anxiety on the Anxiety, Depression, and Mood Scale (ADAMS) were recruited online and offered access to Healthy Mind. Assistance from a nominated supporter was optional. Primary outcomes were symptoms of depression and anxiety (ADAMS). Secondary outcomes were psychological distress (Kessler Psychological Distress Scale [K10]) and functional impairment (World Health Organization Disability Assessment Schedule 2.0 [WHO-DAS]). Outcomes were assessed at baseline, 8 weeks, and 3 months. In total, 80 participants (mean age 27.8, SD 7 y; n=37, 46% female; n=39, 49% identifying as Aboriginal and/or Torres Strait Islander) enrolled; 61% (n=49) nominated a supporter. Data were analyzed using multilevel models with random intercepts for participants.

**Results:**

No significant changes were found in ADAMS depression or anxiety scores from baseline to postintervention or follow-up. Similarly, no significant effects were found for K10 or WHO-DAS scores, except for an improvement in K10 scores between baseline and 3 months when controlling for cognitive functioning (n=15). Having a supporter was associated with lower baseline distress but did not moderate treatment effects. Engagement with Healthy Mind was low; 42.5% (n=34) did not access the program, and among those who did, the median number of completed modules was 7 (IQR 3-11). Greater module completion was associated with slightly higher WHO-DAS scores post intervention.

**Conclusions:**

This trial did not demonstrate significant improvements in mental health or functioning associated with Healthy Mind, likely due to low engagement, reduced statistical power, and the absence of a control group. Nonetheless, the study demonstrates the feasibility of recruiting and retaining people with intellectual disability in fully online trials and highlights the urgent need for strategies to improve engagement, including gamification, personalized content, and integrated social features. Electronic mental health remains a promising avenue for addressing the substantial mental health service gap for people with intellectual disability.

## Introduction

Despite experiencing rates of mental illness 2‐3 times higher than the general population [1], people with intellectual disability intellectual disability face numerous barriers to accessing care [2]. Many health professionals worry they lack the expertise necessary to provide engaging and effective treatment for people with intellectual disability [3,4], and many people with intellectual disability avoid health care due to anxiety or embarrassment [5]. Once someone with intellectual disability does access mental health care, they often receive treatments designed and tested in neurotypical patients that require tailoring to be effective [6]. Diagnostic overshadowing often obscures mental health diagnoses in people with intellectual disability [7], further delaying treatment and leading policymakers to underestimate the need for specialized services. Australia’s National Disability Insurance Scheme excludes mental health care and has been accused of marginalizing people with complex needs [8], and our Statewide Intellectual Disability Mental Health Hubs require a referral, adding more steps to accessing care. To address these barriers, we need a much broader range of services that reflect the needs and wants of people with intellectual disability.

Better and more available mental health services could change the life course of many people with intellectual disability. While life transitions are a source of excitement for many young adults, they can precipitate significant anxiety for those with intellectual disability [9]. Changing social, occupational, and housing situations, coupled with concerns about coping with these changes, can cause significant psychological distress [10] for young adults with intellectual disability. Successful coping during life transitions appears to be an important factor for the long-term functioning of people with intellectual disability [11,12]. With the right mental health support in place, we could see more young people with intellectual disability transition into adulthood with greater happiness and independence.

To realize the many potential benefits of mentally healthy people with intellectual disability, we require better and more accessible mental health programs that boost psychosocial functioning. Greater independence in people with intellectual disability can have significant psychosocial benefits [13]. Being able to participate in daily life as a child can be a buffer against the negative effects intellectual disability can have on quality of life [14]. Promoting autonomy in people with intellectual disability can reduce anxiety and boost problem-solving [12], with increased self-determination associated with long-term well-being [15]. Better mental health for individuals with intellectual disability is also associated with better mental health for carers [16,17], so effective care can benefit families and communities. Given the current scarcity of suitable care [5], innovation is required.

Electronic mental health (eMH) has delivered effective mental health care to millions of people with anxiety and depression [18,19] but remains an underexplored option for people with intellectual disability [20]. While evidence for digital mental health interventions specifically designed for people with intellectual disability remains limited, preliminary studies have shown promise. A recent qualitative systematic review found that adults with intellectual disability generally hold positive views about digital mental health interventions, although accessibility and usability barriers remain significant concerns. However, controlled trials examining clinical effectiveness are lacking, highlighting a critical evidence gap.

eMH is well placed to address many of the recommendations for adapting cognitive behavioral therapy for people with intellectual disability, such as shortened sessions, modified language, highly visual activities, and tools that are easy for carers to use and be involved with [6,21]. Digital modules can be varied in length and interactivity, along with easily incorporated audio assistance. Some digital therapy tools even appear to increase treatment engagement and therapeutic alliance [22], suggesting eMH programs could act as self-guided tools or as adjuncts to traditional face-to-face therapy in blended models of care for people with intellectual disability.

eMH programs must also understand and address the “disability digital divide”—a heterogeneous set of challenges people with intellectual disability face when accessing the internet [23]. Complex language and instructions, hierarchical menus, and multistep processes add significant cognitive load. Videos without captions and text that cannot be resized further complicate their experience [24]. Privacy policies are hard for most users to digest, and legalistic declarations about personal information security are rarely read [25]. Moreover, the absence of inclusive design practices in usability testing involving people with intellectual disability results in tools that do not meet their needs [26]. To be effective, eMH programs must address these issues through simplified language, user-friendly interfaces, and by centering people with intellectual disability and their varied needs in the development process.

Consequently, we conducted a clinical trial to examine whether a self-guided web-based transdiagnostic mental health program (Healthy Mind) could improve the mental health of people with intellectual disability. In this context, “transdiagnostic” refers to interventions that target common psychological processes underlying multiple mental health conditions (eg, anxiety and depression) rather than disorder-specific approaches. “Self-guided” denotes interventions that users complete independently without therapist involvement, though optional support from informal carers or supporters may be available. “Intellectual disability” is characterized by significant limitations in both intellectual functioning (reasoning, learning, and problem-solving) and adaptive behavior (conceptual, social, and practical skills), with onset during the developmental period. In this study, we focused on individuals with borderline to mild intellectual disability, which represents the least severe end of the intellectual disability spectrum.

In conducting this study, we predicted that using Healthy Mind would reduce symptoms of anxiety and depression and improve daily functioning in people aged 16 years and older with borderline-to-mild intellectual disability. This was revised from an original experimental hypothesis, as our study was initially planned as a 2-arm randomized controlled trial (RCT) conducted fully online [27]. At the onset of the COVID-19 pandemic, the Healthy Mind program was released to the public by request of the program funder (Australian Department of Health), unfortunately contaminating the control arm. In response, the study changed to a single-arm uncontrolled trial. While this change limited our ability to draw causal inferences, the single-arm design remained appropriate for examining within-person changes over time and generating preliminary evidence regarding the intervention’s potential effects and implementation feasibility.

## Methods

### Study Design

Although initially designed as a 2-arm RCT [27], the study was converted to a single-arm, uncontrolled design following the public release of the Healthy Mind program due to COVID-19–related circumstances. This shift limited internal validity and causal inference; thus, analyses focused on within-person changes over time rather than between-group comparisons. Results should therefore be interpreted with due caution. At the time of conversion, 3 people had been allocated to the control arm. These participants were offered the opportunity to complete their trial participation and earn full recompense but were excluded from analyses. All participants had full access to the Healthy Mind program after public release.

### Recruitment

#### Eligibility

Participants were eligible if they were Australian residents, aged 16 years or older, self-reported having been diagnosed with borderline or mild intellectual disability, self-reported mild-to-moderate symptoms of depression or anxiety on the Anxiety, Depression, and Mood Scale (ADAMS), and were able to use a computer to access the internet. Intellectual disability diagnosis was self-reported, which was verified by a nominated supporter, where one was available. The supporter’s role was limited to confirming the diagnosis and providing technical and literacy assistance during online tasks; supporters were instructed not to answer on participants’ behalf or influence questionnaire responses. We attempted to collect an estimate of cognitive functioning to further confirm eligibility; however, only 26.25%‐33.75% of the sample were able to complete the assigned tests. For those participants who completed cognitive tests, standardized scores largely fell 1.5‐2 SDs below the normative mean (Table 1), providing objective evidence of impaired intellectual functioning.

We initially planned to mandate that each participant nominate a support person; however, lived experience advisors (LEAs) warned that this may not respect participants’ autonomy and could deter people who preferred to work independently. Therefore, nomination of a support person (hereafter “supporter”) was optional. Consent, screening, and all other data collection were completed online with assistance from the supporter, where applicable.

Participants were ineligible if they reported an intellectual disability diagnosis in the moderate range or above, had medical comorbidities or other functional impairments that might impede trial engagement (eg, impaired vision, hearing, and motor skills), and/or reported severe mental health concerns (severe symptoms of anxiety or depression, severe mood disturbance or mania, suicidal ideation, and psychotic symptoms). We offered ineligible participants resources for seeking alternative mental health support. Any potential participant reporting severe mental health symptoms was referred to their GP for further assessment. Participants reporting acute distress were referred to phone services offering immediate support, including Lifeline, Kids Helpline, and the Suicide Call Back Service.

**Table 1.** Standardized (*z* score) means, SDs, and 95% quantile ranges for scores on Creyos cognitive functioning tasks benchmarked against Creyos age-based norms.

Gender and age range (y)	Digit span		Feature match		Grammatical reasoning		Spatial planning	
	Mean (SD)	95% quantile	Mean (SD)	95% quantile	Mean (SD)	95% quantile	Mean (SD)	95% quantile
Female								
10‐19	−1.77 (0.80)	−2.00 to −1.31	−2.05 (0.36)	−2.21 to −1.73	−2.32 (0.51)	−2.49 to −1.98	−0.94 (0.07)	−0.96 to −0.89
20‐29	−1.65 (0.51)	−2.14 to −0.88	−1.88 (0.85)	−2.21 to −1.73	−2.44 (0.78)	−2.89 to −1.59	−0.62 (1.57)	−1.48 to 2.35
30‐39	−2.13 (1.17)	−2.46 to −1.45	−2.40 (0.69)	−2.97 to −1.65	−2.80 (0.38)	−2.99 to −2.44	−0.78 (0.39)	−1.05 to −0.18
40‐49	−1.00 (1.42)	−1.50 to −0.05	−2.31 (2.09)	−3.05 to −0.90	−1.99 (0.28)	−1.99 to −1.71	−0.62 (1.37)	−1.10 to 0.30
50‐59	—^a^	—	—	—	—	—	—	—
Male								
10‐19	−1.34 (0.97)	−1.68 to −0.69	−0.27 (1.15)	−0.67 to 0.51	−0.88 (0.66)	−1.11 to −0.43	−1.79 (0.52)	−1.98 to −1.44
20‐29	−1.86 (0.49	−2.04 to −1.54	−2.56 (1.28)	−3.01 to −1.70	−3.62^b^	−3.62 to −3.62	0.17 (0.63)	−0.01 to 0.54
30‐39	−1.99 (0.38)	−2.21 to −1.58	−3.01 (0.52)	−3.30 to −2.45	−2.34 (0.31)	−2.47 to 2.08	−1.75^b^	−1.75 to −1.75
40‐49	—	—	—	—	—	—	—	—
50‐59	−2.18^b^	−2.18 to −2.18	−2.07^b^	−2.07 to −2.07	−1.11^b^	−1.11 to −1.11	−0.86^b^	−0.86 to −0.86
Nonbinary^c^								
10‐19	—	—	—	—	—	—	—	—
20‐29	−0.78^b^	−0.78 to −0.78	−0.35^b^	−0.35 to −0.35	−3.33^b^	−3.33 to −3.33	−0.69^b^	−0.69 to −0.69
30‐39	—	—	—	—	—	—	—	—
40‐49	0.00^b^	0.00 to 0.00	−1.42^b^	−1.42 to −1.42	−1.11^b^	−1.11 to −1.11	1.03^b^	1.03 to 1.03
50‐59	—	—	—	—	—	—	—	—

a Not available.

b SD value not available.

c Standardized using female sex and age norms.

#### Power Analysis

For the planned 2-arm design, we estimated a total sample size of 100 participants (50 per group) to achieve 80% power to detect an intervention effect of 0.5 SDs between the control and intervention groups for anxiety and depression symptoms (as measured via the ADAMS) at a 2-tailed α of .05 and accounting for approximately 30% attrition [27]. In response to the contamination of the control arm, we ran 2 additional power analyses. First, we estimated how many participants needed to reach 80% power to detect a within-person treatment effect of 0.5 SDs at a 2-tailed α of .05, accounting for 41.25% attrition at 3 months follow-up (observed attrition rate). We estimated that we needed a total sample size of 200 to reach 80% power. Next, we estimated that for our sample size of 80, we had approximately 58% power to detect a within-person treatment effect of 0.5 SDs. Results should therefore be interpreted with caution and not be interpreted as evidence of intervention ineffectiveness.

#### Recruitment Protocol

Recruitment was open and internet-based. The fully web-based trial was advertised to people aged 16 years and older with intellectual disability and their supporters via Black Dog Institute’s (BDI) social media channels and through promotional activities by our existing partner networks (eg, intellectual disability researchers, clinicians, advocacy groups, and disability support services, some of which had a high number of Aboriginal clients). The recruitment materials described the study as an opportunity to learn new ways of managing difficult thoughts and feelings. Consent, screening, and all other data collection were completed online via an open-access study website built on BDI’s proprietary Research Engine platform, with a supporter where one was nominated.

### Data Quality

Data quality was verified through systematic screening for potentially erroneous or fraudulent responses. Verification criteria included (1) email addresses containing random alphanumeric strings inconsistent with genuine addresses, (2) phone numbers that did not conform to Australian standards, (3) multiple registrations from the same IP address within 10 minutes, (4) identical or near-identical personal details across multiple registrations, and (5) medical history entries unrelated to intellectual disability (eg, repeated use of placeholder text such as “XXX”). Records flagged by these criteria underwent identity verification via phone call. Records that could not be verified or where verification calls revealed inconsistencies were excluded from analyses. A total of 85 registrations were excluded following this verification process. Where fraud was suspected, the participant was notified via the email provided that they were not eligible for the study and offered the resources detailed above. Despite these safeguards, we acknowledge that some fraudulent entries may have remained undetected, representing a limitation of fully online research with vulnerable populations.

### Retention Strategy

To maximize study engagement and retention, participants who completed questionnaires at each measurement occasion received a digital shopping voucher worth Aus $20 (US $14.48) for them and their supporters (where applicable). A scheduled email reminder prompted participants to complete questionnaires. Participants and supporters who did not respond to the scheduled prompt sent by email and subsequent SMS reminder received a phone call reminder from a trial team member. If neither participant nor supporter responded to the phone call reminder, the participant was considered lost to follow-up.

### Final Sample

Baseline demographic variables for our sample are reported in Table 2. Our sample comprised 80 people who reported borderline (n=23, 28.75%) or mild (n=57, 71.25%) intellectual disability living in Australia. Our participants had a mean age of 27.83 (SD 9.34) years, with an approximately even number reporting their sex as female (n=37, 46.25%) and male (n=41, 51.25%), an approximately even number identifying as Aboriginal and/or Torres Strait Islander (n=39, 48.75%) or non-Indigenous (n=41, 51.25%) Australians, and with a slight majority employed (n=47, 58.75%) versus unemployed (n=33, 41.25%). Most participants self-reported their ethnicity as Australian (n=69, 86.25%), their sexuality as heterosexual (n=69, 86.25%), and as having completed formal education to year 12 or above (n=54, 67.5%). Approximately 49 (61.25%) participants reported having a supporter assist them through the study, while 31 (38.75%) did not. Finally, while all participants were asked to complete Creyos [28] online tests of cognitive functioning, only 30 (37.5%) participants returned data for at least 1 of the 4 cognitive tests.

**Table 2.** Baseline sample characteristics (missing data are not included).

Variable	N=80, n (%)
Sex	
Female	37 (46.25)
Male	41 (51.25)
Nonbinary	2 (2.5)
Sexuality	
Bisexual	3 (3.75)
Gay	5 (6.25)
Pansexual	1 (1.25)
Straight	69 (86.25)
Unsure or prefer not to say	2 (2.5)
Indigeneity	
Aboriginal	36 (45)
Aboriginal and Torres Strait Islander	2 (2.5)
Non-Indigenous	41 (51.25)
Torres Strait Islander	1 (1.25)
Ethnicity	
Asian	1 (1.25)
Australian	69 (86.25)
European	5 (6.25)
Indian	1 (1.25)
Middle Eastern	1 (1.25)
Mixed Race	2 (2.5)
White	1 (1.25)
Disability severity	
Borderline	23 (28.75)
Mild	57 (71.25)
Disability etiology	
Chromosomal	20 (25)
Congenital	5 (6.25)
Movement	1 (1.25)
Multiple	12 (15)
Neurodevelopmental	11 (13.75)
Neurological	7 (8.75)
Unspecified	22 (27.25)
Education	
Below year 10	8 (10)
Year 10	18 (22.5)
Year 12	38 (47.5)
Apprenticeship or trade certificate	7 (8.75)
Diploma or other certificate	8 (10)
Undergraduate	1 (1.25)
Employment	
Unemployed	33 (41.25)
Casual	5 (6.25)
Part-time	36 (45)
Full-time paid work	4 (5)
Full-time carer or home duties	2 (2.5)
Student	
Not a student	63 (78.75)
Part-time	8 (10)
Full-time	9 (11.25)
K10^a^	
Likely to be well (<20)	4 (5)
Mild (20-24)	17 (21.25)
Moderate (25-29)	18 (22.5)
Severe (≥30)	30 (37.5)

a K10: Kessler Psychological Distress Scale.

### Intervention

**Figure 1.**
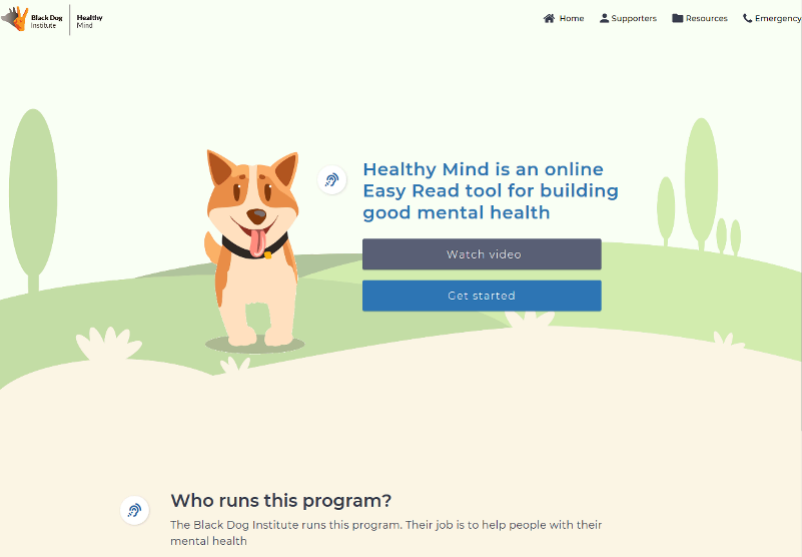
Screenshot of the Healthy Mind landing page.

Healthy Mind is a fully automated, web-based transdiagnostic mental health program designed by researchers, clinicians, user experience experts, and LEAs to meet the needs of people with intellectual disability and developed using funding from the New South Wales Department of Health in Australia (refer to Figures1^3^ for screenshots). All Healthy Mind content was created by researchers and clinicians with expertise in intellectual disability. The first version of the Healthy Mind website was developed by user experience designers, and the website was then refined iteratively based on feedback from LEAs, some of whom were people with intellectual disability and some of whom were carers. Full details are published elsewhere [29], but in brief, LEAs with intellectual disability voiced clear preferences for a simplified website structure, explainer videos for all activities, instructions in both written and audio format, and a “digital guide” to help them use the program. LEAs who were carers requested easy access to resources that can assist with supporting the mental health of a person with intellectual disability.

**Figure 2.**
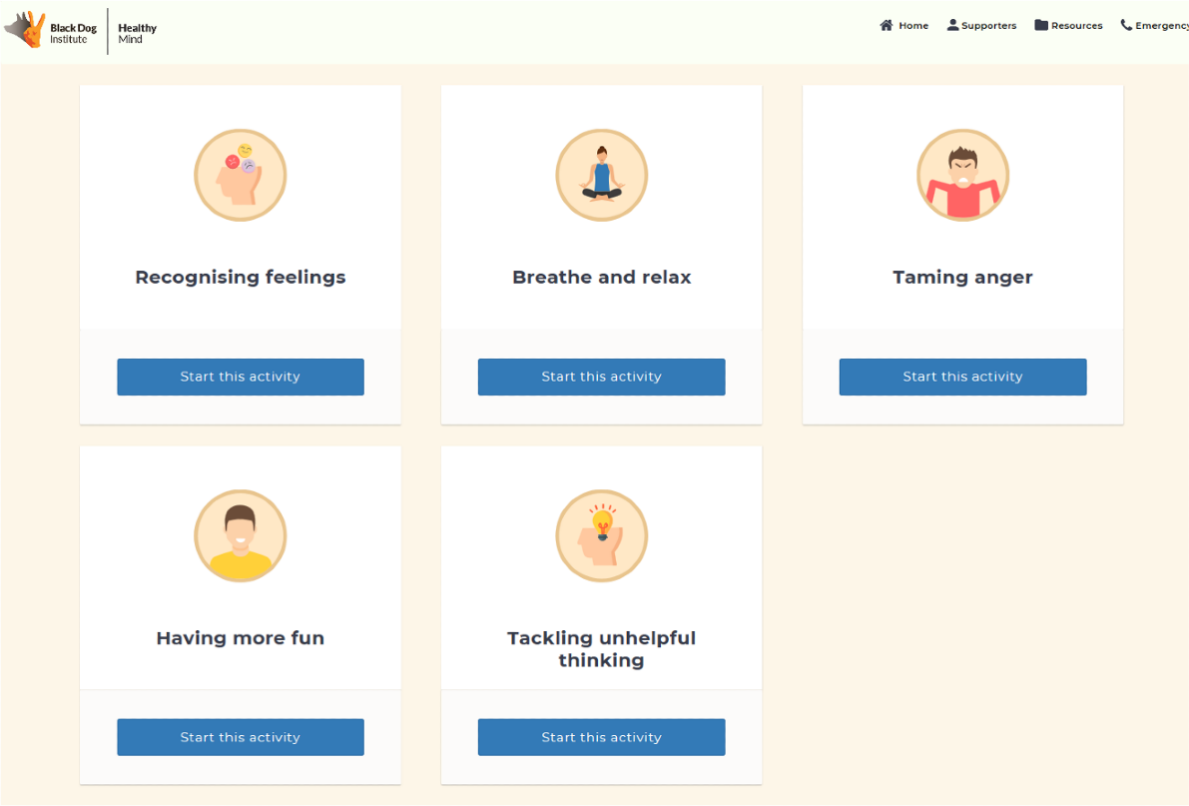
Screenshot of the Healthy Mind dashboard.

**Figure 3.**
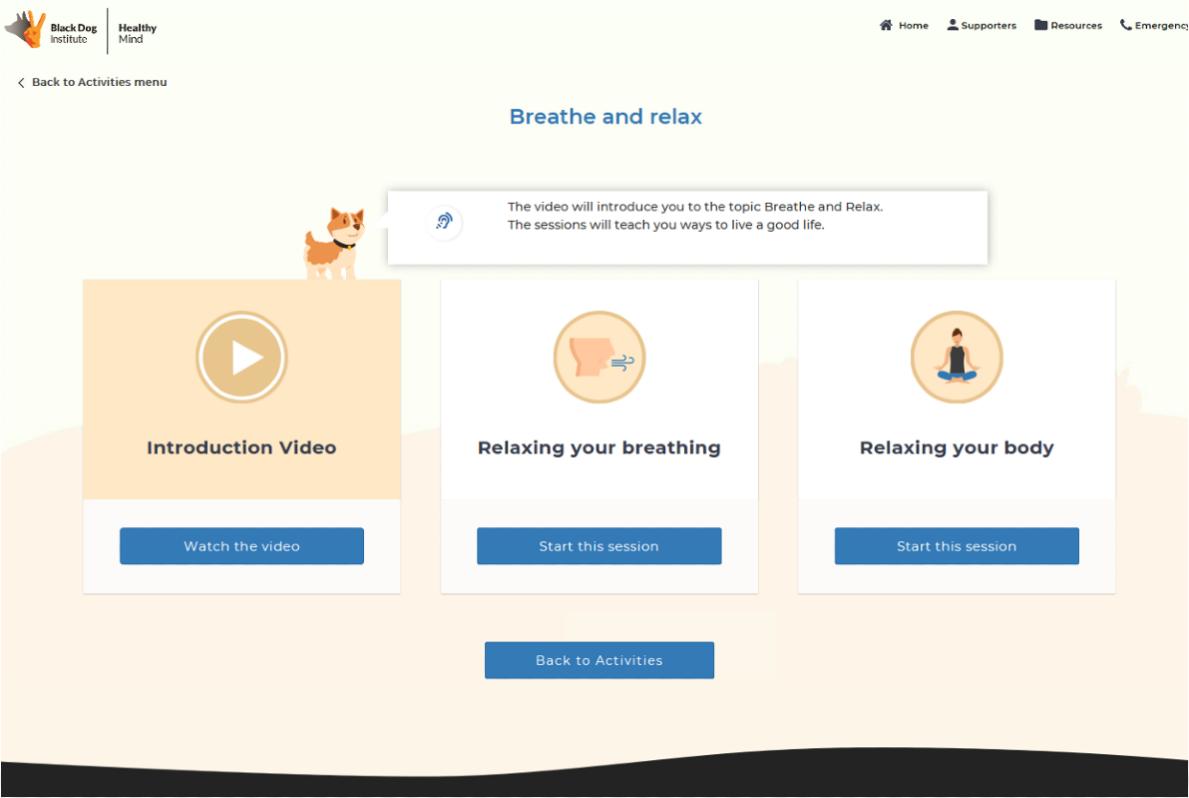
Screenshot of the Healthy Mind “breathe and relax” activity.

Healthy Mind was designed as a self-guided intervention; thus, participants had free access to the full program for the duration of the intervention period, and completion of activities was either self-directed or suggested by a participant’s nominated supporter (no reminders were sent to engage in the program, and no access frequency was prescribed). Participants did not receive any support from the research team to engage with the intervention beyond the aforementioned standardized reminder emails encouraging them to complete the activities within the Healthy Mind program.

The Healthy Mind website contains five interactive activities (Figure 1): (1) “Recognizing feelings,” to help participants name and manage negative emotions such as sadness, anger, or worry; (2) “breathe and relax,” to show participants how to manage emotions using controlled breathing; (3) “taming anger,” to help participants manage frustration and communicate calmly when overwhelmed; (4) “having more fun,” to help participants to plan enjoyable activities across their week; and (5) “tackling unhelpful thinking,” to teach participants cognitive reappraisal of stressful thoughts. Each activity was divided into 2 topics, each comprising the same three steps: (1) “learn about it,” which explained the topic; (2) “watch it” in which participants watched a short video demonstrating the skill; and (3) “do it,” which helped users identify opportunities to use the skill (Figures1^3^). Participants, therefore, had 30 discrete subactivities that they could choose to complete.

All activities were written in Easy Read English using a combination of simple illustrations and photographs to communicate concepts with accompanying audio instructions, interactive activities, and worksheets to download. Videos were brief animations developed specifically for the Healthy Mind program. Site pages kept text to a minimum, with simple icons to direct the user to the site’s audio functions. Links were displayed as large buttons, with descriptions in plain language. As requested by LEAs, an animated dog named Fido acted as a “digital guide” to assist participants in navigating the website. Progress was tracked using a progress bar whereby moving through each step of a module moved Fido closer to a treat on the right-hand side of the page. A separate section labeled “Supporters” contained resources for carers, and the “Emergency” section offered emergency contact options for users who may be experiencing significant distress. Development of the website was completed before the trial, and no updates, downtimes, bug fixes, or content changes occurred during the trial.

During pilot testing with LEAs, we encountered numerous challenges in creating a robust password system for people with intellectual disability, especially due to the differences in how people with intellectual disability use language and memory. Icon-based password systems (eg, a sequence of icons showing a user’s favorite foods) offered inadequate security. Therefore, the Healthy Mind site does not require a login. To assist with user privacy, we designed all activities to never require users to enter personal information and avoided web elements where users might accidentally enter such information (eg, free text fields). Identifiable information collected during the study was only entered into a separate study website, as described.

### Measures

#### Overview

**Figure 4.**
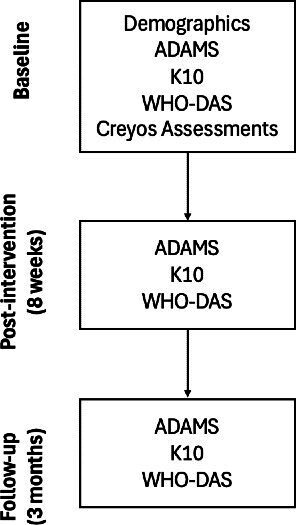
Online measurements completed at each measurement point. ADAMS: Anxiety, Depression, and Mood Scale; K10: Kessler Psychological Distress Scale; WHO-DAS: World Health Organization Disability Assessment Schedule.

Our measurement protocol was kept brief to minimize the attrition common in intellectual disability research [30]. Participants completed all surveys online via the study website as self-report measures. Where participants had a nominated supporter, the supporter was permitted to assist with reading and interpreting questions but was instructed not to answer on the participant’s behalf. This approach prioritized participant autonomy in line with recommendations from our LEAs. Every measurement was included at baseline (ie, including demographics), while participants only completed the outcome scales at postintervention (8 weeks after baseline), and at 3-month follow-up (Figure 4). We used Cronbach α, a measure of internal consistency, as an indicator for the reliability of our outcome measures. For our repeated measures, we calculated within-person (α*^w^*) and between-person (α*^b^*) reliability to account for our data’s nested structure in line with recent recommendations [31]. We noted high rates of participant noncompliance across the study and attrition at postintervention and follow-up. We provide the sample sizes for our primary and secondary outcome measures at each measurement occasion in Table 3.

**Table 3.** Sample sizes for primary and secondary outcome measures across measurement occasions. Percentages indicate amount of total sample (N=80) to complete each measure at each measurement occasion.

	Baseline, n (%)	8 weeks, n (%)	3 months, n (%)
Primary outcomes			
ADAMS^a^ (General Anxiety Subscale)	80 (100)	53 (66.25)	46 (57.5)
ADAMS (Depressed Mood Subscale)	77 (96.25)	53 (66.25)	47 (58.75)
Secondary outcomes			
K10^b^	69 (86.25)	45 (56.25)	41 (51.25)
WHO-DAS^c^	66 (82.5)	47 (58.75)	45 (56.25)

a ADAMS: Anxiety, Depression, and Mood Scale.

b K10: Kessler Psychological Distress Scale.

c WHO-DAS: World Health Organization Disability Assessment Schedule 2.0.

#### Demographics

At baseline, participants were asked to self-report their age, gender identity, ethnicity, Indigenous status, country of birth, state of residence, living situation, relationship status, employment status, level of education, intellectual disability severity, and related diagnosis.

#### ADAMS

Our primary outcome measure was the ADAMS [32], specifically the Depressed Mood and General Anxiety subscales. Each subscale contains 7 items that ask participants to describe the extent to which a given statement is a problem for them on a 4-point Likert-type scale, where 0=“Not a problem” and 3=“Severe problem.” The Depressed Mood subscale uses statements such as, “Sleeps more than normal,” and the General Anxiety subscale uses statements such as “Does not relax or settle down.” In our sample, the General Anxiety subscale demonstrated acceptable within-person reliability (α*^w^*=0.71) and excellent between-person reliability (α*^b^*=0.90), while the Depressed Mood subscale showed poor within-person reliability (α*^w^*=0.51) but excellent between-persons (α*^b^*=0.93) reliability. Although the within-person reliability for the ADAMS Depressed Mood subscale was below the conventional threshold, this measure was retained due to its strong theoretical relevance as an instrument developed specifically for people with intellectual disability and previous evidence of construct validity in similar populations. The low within-person reliability may reflect the adaptation from informant-report to self-report format, and this limitation should be considered when interpreting results for this subscale.

#### Kessler Psychological Distress Scale

The Kessler Psychological Distress Scale (K10) [33] is a 10-item measure of general psychological distress common in Australian clinical practice. Participants rate the extent to which they have experienced a range of mental health symptoms over the preceding 4 weeks on a 5-point Likert-type scale where 1 is “None of the time” and 5 is “All of the time.” In our sample, the reliability of the K10 was acceptable for within-persons (α*^w^*=0.74), and excellent between-persons (α*^b^*=0.93).

#### The World Health Organization Disability Assessment Schedule 2.0

The World Health Organization Disability Assessment Schedule 2.0 (WHO-DAS) [34] measures a person’s disability and general functioning. The WHO-DAS is a 36-item questionnaire assessing participants’ self-reported levels of disability across six life domains that are (1) cognition (6 items; eg, “Concentrating on doing something for ten minutes?”), (2) mobility (5 items; eg, “Standing for long periods such as 30 min?”), (3) self-care (3 items; eg, “Washing your whole body?”), (4) getting along with others (5 items; eg, “Dealing with people you do not know?”), (5) daily life activities (9 items; eg, “Taking care of your household responsibilities?”), and (6) participating in society (8 items; eg, “How much of a problem did you have because of barriers or hindrances in the world around you?”). Participants reported the level of difficulty they have experienced in the past 30 days for each item on a 5-point Likert-type scale where 1 is “None” and 5 is “Extreme or cannot do.” WHO-DAS domain scores were computed using the “item-response-theory” method. Because the largest minority of participants reported not having an occupation (eg, employment or studying), we scored the WHO-DAS without items that measure participants’ functioning at work. For the overall scale, reliability in our sample was good within-persons (α*^w^*=0.89), and excellent between-persons (α*^b^*=0.97).

#### Cognitive Assessment

Participants were asked to complete four cognitive assessment tasks via the Creyos [28] online platform: (1) The digit span task estimated participants’ ability to remember a sequence of numbers (working memory), (2) the grammatical reasoning task estimated participants’ ability to use written information to draw conclusions (verbal reasoning), (3) the spatial planning task measured participants’ ability to use visual information to plan ahead (spatial reasoning), and (4) the feature match tested participants’ ability to focus their attention on specific features of an image while ignoring others (focused attention). Despite all participants receiving the same test battery, there was significant heterogeneity in which tasks were completed by each participant, if any. Respectively, 25, 27, 21, and 27 participants completed the above tasks.

#### Treatment Engagement

Treatment engagement was measured via user-level web analytics that quantified whether a user accessed a particular page or opened a particular activity. Time-on-task metrics were not available due to limitations of our analytics technology. Qualitative feedback was not sought.

### Procedure

**Figure 5.**
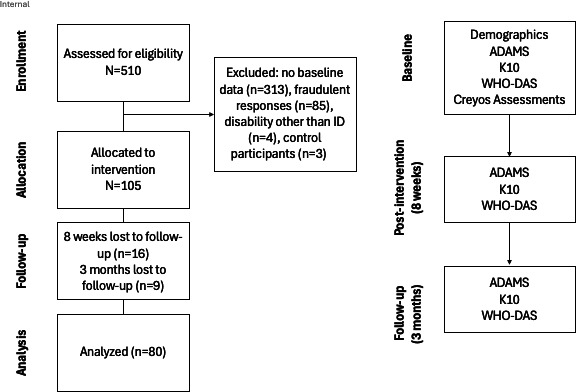
CONSORT (Consolidated Standards of Reporting Trials) diagram of participant flow through study. ADAMS: Anxiety, Depression, and Mood Scale; ID: intellectual disability; K10: Kessler Psychological Distress Scale; WHO-DAS: World Health Organization Disability Assessment Schedule 2.0

Participant consent, screening, and assessment took place entirely online using Easy Read materials designed in consultation with LEAs to maximize accessibility. Written information was accompanied by audio explanations to accommodate diverse literacy levels. While this approach was designed to reduce barriers to participation and respect participant autonomy, it did not include direct verbal confirmation of understanding with research staff. Potential participants and their supporter (where applicable) started by completing the online screening questionnaire to establish eligibility. Ineligible participants were directed to a website page advising them of their ineligibility and offering support service information. Eligible participants then provided informed consent online before being directed to complete the baseline questionnaires. All self-report data were captured electronically via standardized, self-report questionnaires that were completed via the Healthy Mind study website. Upon receipt of baseline questionnaire data, participants received (1) an email requesting they complete a Creyos [28] fully online cognitive assessment; and (2) an email directing them to the Healthy Mind website (refer to Intervention section) with which they were encouraged to engage freely, either independently or with a supporter. Participants were subsequently asked via email to complete follow-up assessments at 8 weeks (post intervention) and 3 months (follow-up). At each assessment point, the study website automatically issued a unique link to the study questionnaire via email. Figure 5 illustrates the flow of participants through the study, noting attrition at each follow-up occasion.

### Ethical Considerations

The Healthy Mind study protocol and materials were approved by the Human Research Ethics Committee at the University of New South Wales (HC190393). The consent process used Easy Read materials and audio explanations developed in consultation with LEAs to ensure accessibility. All identifiable data were stored on encrypted servers accessible only to approved research staff. The Healthy Mind intervention website was designed following Web Content Accessibility Guidelines 2.1 principles to accommodate diverse cognitive and sensory needs, including simplified navigation, minimal text, audio support, and large clickable elements.

### Data and Analyses

#### Data Collection

All questionnaire data were entered online via the secure study website and captured by the BDI Research Engine, a proprietary online study management system that stores encrypted data on servers located at the University of New South Wales, Sydney, and is only accessible by approved research staff. Cognitive function data were collected electronically using Creyos [28] web-based assessments administered via the Creyos website. All data were encrypted during transmission to, and storage on, Amazon’s AWS EC2 servers in Oregon, United States. Once stored, data were accessible by only PB, VR, and WG on the password-protected Creyos [28] platform. The only other individuals with access to the cognitive assessment data were Creyos administrators who accessed the BDI account to assist with technical issues.

#### Data Analysis

All analyses were performed in R-Studio version 4.2.2 (Posit). We wrangled the data using the *tidyverse* suite of packages [35].

##### Preliminary Analyses

Before our main analyses, we computed within- and between-person means, SDs, and correlations using the *psych* package [36]. We computed within- and between-person statistics to account for our data’s nested, longitudinal structure. We used the *lme4* package [37] to compute intraclass correlations (ICC) as per the formula for ICC(1) [38] to determine whether data were suitable for linear mixed-effect modeling.

##### Primary (Intention-To-Treat) Analyses

To establish if the intervention was effective, we ran a series of multilevel models using the *lme4* package [37,38] that included a random intercept for participant to account for our data’s nested structure (ie, repeated measures nested within individuals) and used a maximum likelihood estimator to manage missing data. Our models tested the main effect of time (3 levels—baseline, 8 wk, and 3 mo) on our primary (the ADAMS Depressed Mood and General Anxiety subscales) and secondary (WHO-DAS 2.0 and K10 scales) outcome measures making three comparisons: (1) whether the intervention had significantly decreased (ie, improved) outcome measures from baseline to postintervention; (2) whether the intervention had significantly decreased outcome measures from baseline to 3 months follow-up; and (3) whether any improvements at 8 weeks were maintained by 3 month follow-up.

##### Covariate Analyses

To explore whether cognitive functioning accounted for outcome variance over time (ie, impacted the effect of the intervention), we reran the above models, adding in our measures of cognitive function (digit span, feature match, grammatical reasoning, and spatial planning) as covariates.

##### Secondary Analyses

As a follow-up to our main analyses, we used multilevel models to test whether participation in Healthy Mind (operationalized as the number of modules a participant completed) and/or supporter support moderated the relationship between intervention and our primary and secondary outcomes. To do this, we included an interaction term between intervention time point, modules completed (a continuous variable ranging from 0 to 30), and whether participants had a supporter (a Boolean variable indicating “nominated supporter” or “no nominated supporter”).

## Results

### Descriptive Statistics

Tables4  5 summarize our descriptive statistics, ICCs, and within- and between-person correlations. Our ICCs for the Depressed Mood and General Anxiety subscales indicated that 54% and 46% of the variance was between-persons, respectively (with the remaining 46% and 54% being within-persons). For K10 and WHO-DAS scores, 54% and 55% of variance was between-persons, respectively. ICCs above 0.40 indicate sufficient between-person variance to warrant multilevel analysis, suggesting our data were suitable for linear multilevel modeling [39]. Finally, our Pearson correlations indicated our primary and secondary outcome variables were moderately-to-strongly correlated, both time point-to-time point (within-person) and on average (between-persons). These correlations suggest that as self-reported functional impairments increased, so did symptoms of anxiety, depression, and psychological distress (and vice versa). The participant flow through the study is shown in Figure 5.

Table 1 presents standardized scores for the Creyos [28] cognitive subtests with sex-based norms represented by 95% quantiles. As expected, our participants’ standardized scores on each cognitive functioning measure were largely between 1.5 and 2 SDs from the normative mean for their age and sex group, suggesting impaired cognitive functioning. However, the number of participants who completed the Creyos [28] cognitive assessment was lower than expected. Of 80 participants, 21 (26.25%) completed the digit span task, 27 (33.75%) completed the grammatical reasoning and feature match tasks, and 25 (31.25%) completed the spatial planning task.

**Table 4.** Means, within and between-person SDs, and intraclass correlations of primary (anxiety and depression) and secondary (distress and disability) outcome variables. Within denotes a within-person statistic, while between denotes a between-person statistic.

Variable	Mean (SD)		ICC^a^
Within	Between
ADAMS^b^ (General Anxiety Subscale)	9.43 (2.60)	9.43 (3.85)	0.46
ADAMS (Depressed Mood Subscale)	8.40 (2.33)	8.40 (3.73)	0.54
K10^c^	28.14 (3.43)	28.14 (5.94)	0.54
WHO-DAS^d^	47.55 (10.47)	47.55 (17.48)	0.55

a ICC: intraclass correlations.

b ADAMS: Anxiety, Depression, and Mood Scale.

c K10: Kessler Psychological Distress Scale.

d WHO-DAS: World Health Organization Disability Assessment Schedule 2.0.

**Table 5.** Pearson correlation matrix of primary (anxiety and depression) and secondary (distress and disability) outcome variables. Correlations below the diagonal of the matrix indicate pooled within-person correlations, while those on above the diagonal indicate between-person weighted mean correlations.

Variable	1	2	3	4
ADAMS^a^ (General Anxiety Subscale)				
*r*	—^b^	0.73	0.35	0.61
*P* value	—	.01	.03	.02
ADAMS (Depressed Mood Subscale)				
*r*	0.52	—	0.47	0.64
*P* value	.02	—	.02	.01
K10^c^				
*r*	0.36	0.35	—	0.36
*P* value	.03	.02	—	.03
WHO-DAS^d^				
*r*	0.50	0.40	0.38	—
*P* value	.01	.04	.02	—

a ADAMS: Anxiety, Depression, and Mood Scale.

b Not applicable.

c K10: Kessler Psychological Distress Scale.

d WHO-DAS: World Health Organization Disability Assessment Schedule 2.0.

### Primary Analyses

#### Primary Outcome Variables

**Figure 6.**
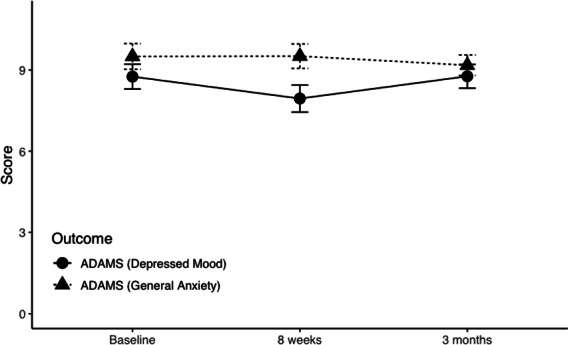
Line graph of Anxiety, Depression, and Mood Scale depressed mood and general anxiety scores across at baseline, and 8 weeks and 3 months follow-up. Error bars represent SEs. ADAMS: Anxiety, Depression, and Mood Scale.

Figure 6 illustrates the change in mean levels of our primary outcome variables, the ADAMS Depressed Mood and General Anxiety scales, across our study time points, with Table 6 summarizing our multilevel models for these subscales. For both of our primary outcome variables, we found no treatment effect for any of the time point comparisons.

**Table 6.** Pairwise effects of intervention predicting primary outcome variables.

Outcome	Estimate^a^ (SE)	95% CI	*z* score	*P* value
ADAMS^b^ (Depressed Mood)				
Intercept	8.74 (0.46)	7.84 to 9.64	19.08	<.001
Baseline to 8 weeks	−0.90 (0.51)	−2.09 to 0.29	−1.77	.08
Baseline to 3 months	−0.24 (0.53)	−1.48 to 1.01	−0.44	.66
8 weeks to 3 months	0.66 (0.56)	−0.65 to 0.66	1.18	.46
ADAMS (General Anxiety)				
Intercept	9.50 (0.43)	8.57 to 10.43	20.11	<.001
Baseline to 8 weeks	−0.01 (0.58)	−1.36 to 1.34	−0.10	.99
Baseline to 3 months	−0.36 (0.61)	−1.78 to 1.07	−0.58	.83
8 weeks to 3 months	−0.35 (0.65)	−1.87 to 1.17	−0.54	.85

a Estimates are unstandardized.

b ADAMS: Anxiety, Depression, and Mood Scale.

#### Secondary Outcome Variables

**Figure 7.**
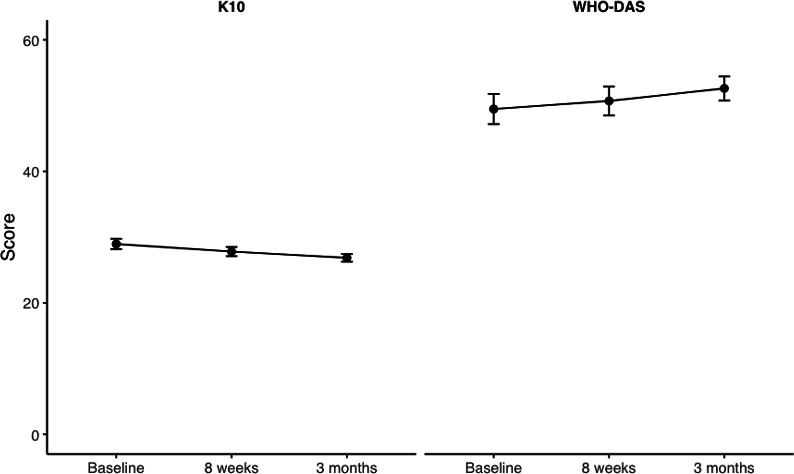
Line graph of self-report disability and psychological distress at baseline, and 8 weeks and 3 months follow-up. Error bars represent SEs. K10: Kessler Psychological Distress Scale; WHO-DAS: World Health Organization Disability Assessment Schedule 2.0.

Figure 7 illustrates the mean change in participants’ scores on our secondary outcome variables (the K10 and WHO-DAS) across the study time points. Table 7 summarizes the findings from our multilevel models. As with our primary outcomes, we found no significant effect overall for either model or for any of the pairwise comparisons.

**Table 7.** Pairwise effects of intervention predicting secondary outcome variables.

Outcome	Estimate^a^ (SE)	95% CI	*z* score	*P* value
K10^b^				
Intercept	28.93 (0.76)	27.44 to 30.43	37.89	<.001
Baseline to 8 weeks	−1.66 (0.89)	−3.74 to 0.43	−1.86	.25
Baseline to 3 months	−1.69 (0.93)	−3.88 to 0.49	−1.82	.16
8 weeks to 3 months	−0.04 (0.97)	−2.831 to 2.24	−0.04	.99
WHO-DAS^c^				
Intercept	49.48 (2.22)	45.14 to 53.83	22.34	<.001
Baseline to 8 weeks	−0.92 (2.38)	−6.50 to 4.65	−0.39	.92
Baseline to 3 months	0.37 (2.50)	−5.50 to 6.25	0.15	.99
8 weeks to 3 months	1.30 (0.65)	−4.91 to 7.50	2.65	.88

a Estimates are unstandardized.

b K10: Kessler Psychological Distress Scale.

c WHO-DAS: World Health Organization Disability Assessment Schedule 2.0.

### Covariate Analyses

The addition of our measures of cognitive function did not affect our results for the ADAMS (General Anxiety), ADAMS (Depressed Mood), or WHO-DAS scales. However, when holding cognitive functioning constant, we found a significant improvement in K10 scores between baseline and 3 months follow-up (n=15, *b*=−6.61, SE 2.55, *t*_12.44_=−2.47; *P*=.02). As previously mentioned, a limited number of participants had valid cognitive functioning data. As such, we are underpowered to make robust claims regarding the associations of our intervention on psychological distress when holding cognitive functioning constant.

### Secondary Analyses

#### Supporter Effects

We did not find significant main effects for having a supporter or time, nor their interaction when predicting General Anxiety and Depressed Mood (*P* values>.05), suggesting that supporters did not influence any effect the intervention may have had on our primary outcomes. For our secondary outcomes, we found a significant main effect for supporter on K10 scores and a significant interaction between supporter and time for WHO-DAS. Specifically, we found that participants with a nominated supporter had significantly higher K10 scores compared with those without a nominated supporter at baseline (*b*=3.76, SE 1.53, *z* score=2.46; *P*=.04), indicating greater psychological distress among participants who sought supporter involvement. Conversely, we found that participants with a supporter reported significantly greater WHO-DAS scores post intervention than did participants without a supporter post intervention (*b*=−18.29, SE 7.01, *z* score=−2.61; *P*=.03). Finally, we found no significant effect of supporter in improving participant engagement with Healthy Mind (*b*=−2.31, SE 2.34, z score=−0.99; *P*=.38), suggesting similar levels of engagement with the intervention for participants with and without a supporter.

#### Engagement With Healthy Mind

Overall, engagement with Healthy Mind was low. Participants completed a mean of 2 of 10 (SD 3.23, ranging from 0 to 10) topics accessed and a mean of 7 of 30 (SD 10.18, ranging from 0 to 30) modules. A large minority of participants (n=34*,* 42.5%) did not engage with the Healthy Mind program at all. Among participants who did engage, 57.5% (n=46) did not complete any of the 10 available topics.

Regarding the multilevel models regressing our outcome measures on treatment engagement, time, and their interaction, we did not find any significant main effects for engagement with Healthy Mind, nor a significant interaction between engagement and time for either the ADAMS Depressed Mood or General Anxiety subscales, or K10 scores (*P* values>.05). We found a significant interaction between number of modules completed, time, and WHO-DAS scores, such that participants who completed a greater number of modules in Healthy Mind self-reported significantly greater disability severity post intervention than those who completed fewer modules (*b*=0.50, SE 0.25, *z* score=2.02; *P*=.05). This effect was significant but small, with only a 0.5-point increase in WHO-DAS scores for every module completed at 8 weeks post intervention.

## Discussion

### Overview

Our single-arm web-based trial aimed to evaluate whether a self-guided web-based transdiagnostic mental health program (Healthy Mind) could reduce symptoms of anxiety and depression in people with intellectual disability. Our descriptive, correlational, and neuropsychological data suggest we were successful in recruiting a sample of people with measurable cognitive impairments and elevated symptoms of anxiety and depression that worsen with increased functional impairment. However, we failed to find an effect of Healthy Mind on anxiety or depression symptoms, or for any other outcome variable.

### Principal Results

Our intention-to-treat analyses included all 80 enrolled participants with an attrition rate of 36.25% at 8 weeks (n=51 retained) and 41.25% at 3 months (n=47 retained). Combined with the estimated 58% power for detecting medium effects, these factors substantially limited our ability to detect true intervention effects if they existed. Given our low statistical power, unexpected changes to trial design, and poor treatment engagement, we cannot attribute the lack of effect to the intervention, although this remains a possible explanation. Due to the public release of the intervention mid-trial and subsequent contamination of the planned control arm, we could not compare participants accessing Healthy Mind to individuals who did not have access to the program. Without a control group, any observed changes (or lack thereof) cannot be attributed to the intervention with confidence, as they may reflect natural symptom fluctuation or external factors, such as the COVID-19 pandemic context. Observed trends should therefore be interpreted as exploratory rather than confirmatory. Moreover, participants’ use of the program was low. Approximately 42% of participants did not access the program at all, and of those who did, less than half completed any 1 topic. As a result, participants likely did not engage sufficiently with the program to receive any intended benefit. A larger, multiarm study with robust intervention engagement is needed to determine if Healthy Mind can produce its intended benefits.

Nonetheless, some curious findings emerged from our analyses. When cognitive function was held constant, we saw a significant improvement in K10 scores across the study, although not in intellectual disability–specific measures of anxiety or depression. This may reflect a problem with convergent validity. The K10 was collected as a secondary outcome because it is commonly used as a mental health screener in Australian primary care, but mainstream measures do not always capture what mental health means to people with intellectual disability [40]. We found a difference in K10 scores between participants with and without supporters at baseline, with this effect disappearing 8 weeks post intervention and 3 months follow-up. It is possible that participants with identified support at the beginning of the trial felt somewhat less distressed, potentially reflecting support generally being more available to them in daily life. The counterintuitive finding that participants who completed a greater number of modules reported significantly greater disability severity post intervention may reflect engagement bias (participants experiencing greater functional impairment may have been more motivated to engage with the intervention) but may also reflect statistical noise, given the small effect size. These statements are, of course, speculative and should be viewed as hypotheses for future research.

Regarding our measures of functional impairment, participants with a supporter and participants who completed a greater number of modules each reported greater impairment after treatment, albeit with quite small effects. Given that the intervention began at the start of the COVID-19 pandemic, participants who required more support before the pandemic may have been more severely impacted by it, and those with higher distress may have been motivated to engage more with the treatment but did not receive any benefit. These findings are difficult to interpret and likely require more data to understand.

### Limitations

This study had several limitations that should be considered when interpreting findings. Regarding study design and sampling, the conversion from RCT to a single-arm design following control arm contamination limited internal validity and causal inference. Selection bias likely affected our study, as participants may have been especially motivated to learn mental health skills and engage with an eMH program designed specifically for people with intellectual disability. Relatedly, they may have carers who are similarly motivated. However, as Healthy Mind is freely available to all Australians, a sample of motivated individuals likely reflects naturalistic uptake of self-help mental health programs.

Despite our deliberately simple protocol, option for a supporter, and automated program reminders, we experienced low engagement, suggestive of the problems with adherence and attrition often observed in clinical trials that include people with intellectual disability [30]. The fully online consent process, while designed to maximize accessibility, did not include direct conversation with research staff to confirm participant understanding. Future studies might consider incorporating brief phone or video calls during the consent process to verify comprehension and provide an opportunity to answer questions, which may also serve to enhance subsequent engagement with the intervention.

Regarding measurement, the ADAMS was originally developed and validated as an informant-report measure rather than a self-report instrument. While our decision to use it as a self-report measure was guided by LEAs who emphasized the importance of participant autonomy and self-determination, this adaptation may have affected measurement validity. The multiple response options and complex wording of some items may have posed comprehension difficulties for some participants, potentially contributing to the low within-person reliability observed for the Depressed Mood subscale. Similarly, while the K10 is a recommended measure in the NSW Health Intellectual Disability Mental Health Core Competency Framework, future research should seek a comprehensive validation of this measure in people with intellectual disability.

### Comparison With Previous Work

The challenges and learnings from this study bear discussion as they may help others create intellectual disability–specific mental health programs. Disappointingly, the trial was a significant target for fraudulent participation, characterized by false email addresses and phone numbers, along with repeated patterns of dummy reporting in medical history (eg, repeated report of “XXX” as the etiology of intellectual disability). This is a reminder of the risks that people with intellectual disability face online and a threat to research validity that must be overcome, ideally via digital health platforms that can detect and discourage fraud. Measures of cognitive functioning also posed a challenge. Online cognitive data collection was so inconsistent that the link between cognitive function and online engagement with a mental health program could not be effectively explored, but remains an important topic for future study.

The challenge of engagement continues to vex digital mental health [41], but emerging research suggests several avenues for boosting engagement with programs, like Healthy Mind, which future studies could explore. Blended care that combines digital and person-to-person care components may increase engagement in technology-based care for people with intellectual disability. Imagery and audio seem to be essential elements of digital design for some people with intellectual disability [42]. Gamification is enhanced in mainstream digital mental health and may drive better engagement for people with intellectual disability [43]. Digital activities that encourage mental imagery and the use of avatars seem to assist those with a range of cognitive impairments, which may translate into more engaging support programs for people with intellectual disability [44]. Data suggest that people with intellectual disability primarily use internet-connected technology for social support [45], which can translate into real benefits to well-being [46]. Future programs like Healthy Mind should consider incorporating a bespoke social networking function that uses accessible written language and takes into account the online safety of people with intellectual disability [47].

Incorporating gamified and social components may enhance intrinsic motivation and maintain engagement, consistent with self-determination theory by Deci and Ryan [48]. According to self-determination theory, autonomy, competence, and relatedness are fundamental psychological needs that drive engagement. Gamification elements (badges, progress tracking, and rewards) may support competence needs, while social features may address relatedness needs. Future programs should consider how these theoretical principles can be operationalized for people with intellectual disability while maintaining accessibility and safety.

### Conclusions

Despite not being able to establish the efficacy of Healthy Mind, our trial represents an example of the innovation urgently needed in mental health care for people with intellectual disability. Access to mental health support remains a substantial challenge in intellectual disability, and eMH could play an important role in improving the range and quality of care available to this group. We must address the barriers and design services based on known facilitators of uptake and well-being, such as dignity, collaboration, and sincere effort to personalize care to the needs of people with intellectual disability [5]. If we can create a broader range of mental health services that reflect the needs and wants of people with intellectual disability, we can help people with intellectual disability create the healthy mind and happy life they deserve.

## Supplementary material

10.2196/82246 Checklist 1 CONSORT EHEALTH checklist.

## Abbreviations

ADAMS: Anxiety, Depression, and Mood Scale
BDI: Black Dog Institute
eMH: electronic mental health
ICC: intraclass correlation
K10: Kessler Psychological Distress Scale
LEA: lived experience advisor
RCT: randomized controlled trial
WHO-DAS: World Health Organization Disability Assessment Schedule 2.0
