# Supplementary material for: A Self-Guided Web-Based Transdiagnostic Mental Health Program for People With Intellectual Disability: Single-Arm Trial
Source: JMIR Form Res. 2026 Jun 11;10:e82246. doi: 10.2196/82246 (PMC13256486; doi:10.2196/82246)
Supplement: Checklist 1 [file formative-v10-e82246-s001.pdf]

CONSORT-EHEALTH (V.1.6.1) Checklist Compliance

Manuscript: *A Self-Guided Web-Based Transdiagnostic Mental Health Program for People With Intellectual Disability: Single-Arm Trial*

Section / Topic	Item No.	CONSORT Checklist Item	EHEALTH Extension	Page(s)	Comment
**TITLE & ABSTRACT**					
**Title**	1a-i	Identification as a randomised trial in the title	Identify the mode of delivery in the title (e.g. "web-based")	1	Title includes "Web-Based" and "Self-Guided"; identifies target group (intellectual disability)
**Title**	1a-ii		Mention non-web-based components or co-interventions in title	1	Not applicable – intervention is fully web-based with no co-interventions
**Title**	1a-iii		Mention primary condition or target group in the title	1	Title identifies "People With Intellectual Disability"
**Abstract**	1b-i	Structured summary of trial design, methods, results, conclusions	Mention key features/functionalities of the intervention in abstract	1–3	Abstract describes Healthy Mind as self-guided, web-based, transdiagnostic program; mentions ADAMS, K10, WHO-DAS
**Abstract**	1b-ii		Clarify level of human involvement in abstract	1–2	Abstract states "self-guided" and notes supporter assistance was optional
**Abstract**	1b-iii		Open vs closed, web-based vs face-to-face assessments in abstract	2	Abstract states participants were "recruited online"; outcomes assessed via self-report questionnaires
**Abstract**	1b-iv		Results in abstract must contain use data	2–3	Abstract reports engagement: 42.5% did not access program; median 7 of 30 modules completed
**Abstract**	1b-v		Conclusions for negative trials: discuss primary outcome and uptake	3	Abstract discusses negative findings and attributes them to low engagement, reduced power, and absence of control group
**INTRODUCTION**					
**Background & objectives**	2a-i	Scientific background and explanation of rationale	Describe the problem and the type of system/solution	4–7	Introduction describes barriers to mental health care for people with intellectual disability; positions eMH as a solution
**Background & objectives**	2a-ii		Scientific background, rationale: what is known about the system	6–7	Reviews existing evidence for digital mental health interventions for people with intellectual disability; discusses disability digital divide
**Objectives**	2b	Specific objectives or hypotheses	No eHealth-specific additions	7–8	Hypotheses stated: predicted Healthy Mind would reduce anxiety/depression and improve functioning; notes revision from original RCT design
**METHODS**					
**Trial design**	3a	Description of trial design including allocation ratio	No eHealth-specific additions	8–9	Single-arm uncontrolled design described; explains conversion from planned RCT due to COVID-19 contamination
**Trial design**	3b-i	Important changes to methods after trial commencement	Bug fixes, downtimes, content changes	8, 16–17	Describes conversion from RCT to single-arm design; states no updates, downtimes, bug fixes or content changes occurred during the trial

CONSORT-EHEALTH Checklist Compliance Table

**Participants**	4a-i	Eligibility criteria for participants	Computer/Internet literacy as eligibility criterion	9	States participants needed to be "able to use computer to access the internet"
**Participants**	4a-ii		Open vs closed, web-based vs face-to-face assessments	9–11	Recruitment was open and internet-based; consent, screening and all data collection completed online; email confirmation used
**Participants**	4a-iii		Information given during recruitment	11	Describes recruitment materials and study description; consent process described under Ethical Approval (p. 26)
**Participants**	4b-i	Settings and locations where data were collected	Report if outcomes were self-assessed through online questionnaires	20–21	States all surveys completed online via study website as self-report measures
**Participants**	4b-ii		Report how institutional affiliations are displayed	11	Black Dog Institute identified as the recruiting organisation with its social media channels and partner networks
**Interventions**	5-i	Interventions for each group with sufficient detail for replication	Mention names, credentials, affiliations of developers/sponsors	15	Describes researchers, clinicians, UX experts, and lived experience advisors as developers; funding from NSW Dept of Health
**Interventions**	5-ii		Describe history/development process and formative evaluations	15	References published feasibility study [29]; describes iterative refinement with LEAs including usability feedback
**Interventions**	5-iii		Revisions and updating: mention date/version of application	16–17	States development completed prior to trial; no updates, downtimes, bug fixes or content changes during trial
**Interventions**	5-iv		Quality assurance methods	–	Not applicable
**Interventions**	5-v		Ensure replicability (source code, screenshots, flowcharts)	17–18	Screenshots of Healthy Mind provided (Figures 2–4); detailed description of modules and activities
**Interventions**	5-vi		Digital preservation: provide URL, archive intervention	11	URL provided (healthymind.blackdoghealth.org.au); no archiving explicitly mentioned
**Interventions**	5-vii		Access: describe how participants accessed the application	15–16, 18	Describes free access, self-guided use, no login required; no payment; open access after public release
**Interventions**	5-viii		Describe mode of delivery, features, theoretical framework	15–17	Describes web-based delivery, five interactive activities based on CBT principles, Easy Read format, audio, animations, digital guide (Fido)
**Interventions**	5-ix		Describe use parameters (intended doses, timing)	15–16	States participants had free access; completion self-directed or suggested by supporter; no prescribed frequency
**Interventions**	5-x		Clarify level of human involvement	15–16	Fully automated, self-guided; no therapist involvement; optional supporter assistance
**Interventions**	5-xi		Report any prompts/reminders used	12, 16	Email reminders sent to complete questionnaires; no reminders sent to engage with the program itself
**Interventions**	5-xii		Describe any co-interventions (incl. training/support)	15–16	No co-interventions; supporter role limited to technical/literacy assistance; separate supporter resources section on website
**Outcomes**	6a-i	Pre-specified primary and secondary	If online questionnaires, describe validation for online use	20–24	Describes ADAMS, K10, WHO-DAS with reliability data; all administered online as self-report; notes ADAMS

CONSORT-EHEALTH Checklist Compliance Table

		outcome measures			originally validated as informant-report
**Outcomes**	6a-ii		Describe whether/how "use" was defined/measured	24–25	Treatment engagement measured via user-level web analytics (page access, activity completion); time-on-task not available
**Outcomes**	6a-iii		Describe whether/how qualitative feedback was obtained	25	States qualitative feedback was not sought
**Outcomes**	6b	Changes to trial outcomes after commencement	No eHealth-specific additions	–	Not explicitly reported; implied no changes to outcomes
**Sample size**	7a-i	How sample size was determined	Describe whether expected attrition was taken into account	10–11	Original power analysis accounted for 30% attrition; revised analysis used observed 41.25% attrition rate
**Sample size**	7b	Interim analyses and stopping guidelines	No eHealth-specific additions	–	Not applicable
**Randomisation**	8a	Method used to generate random allocation sequence	No eHealth-specific additions	–	Not applicable – converted to single-arm design
**Randomisation**	8b	Type of randomisation; details of restriction	No eHealth-specific additions	–	Not applicable – converted to single-arm design
**Allocation concealment**	9	Mechanism for allocation concealment	No eHealth-specific additions	–	Not applicable – converted to single-arm design
**Implementation**	10	Who generated allocation, enrolled, and assigned participants	No eHealth-specific additions	–	Not applicable – converted to single-arm design
**Blinding**	11a-i	Who was blinded after assignment	Specify who was blinded and who was not	8–9	Single-arm unblinded design acknowledged; participants were aware of the intervention
**Blinding**	11a-ii		Discuss whether participants knew which intervention was of interest	–	Not applicable – no comparator group
**Blinding**	11b	Similarity of interventions		–	Not applicable – single-arm design
**Statistical methods**	12a-i	Statistical methods for comparing groups	Imputation techniques for attrition/missing values	27–28	Multilevel models with maximum likelihood estimator to manage missing data; random intercept for participant; intent-to-treat approach
**Statistical methods**	12b	Methods for additional analyses	No eHealth-specific additions	27–28	Covariate analyses with cognitive function; moderation analyses for supporter and engagement
**Ethics**	X26-i	Ethics committee approval	Comment on ethics committee approval	26	HREC approval from UNSW (HC190393); registered with ANZCTR (ACTRN12620000113954)
**Ethics**	X26-ii		Outline informed consent procedures	25–26	Online consent using Easy Read materials with audio explanations; developed with LEA input; limitations of online-only consent acknowledged
**Ethics**	X26-iii		Safety and security procedures incl. privacy	18, 26	No login required for privacy; no personal information entered in activities; data encrypted on UNSW servers; WCAG 2.1 compliance
**RESULTS**					

CONSORT-EHEALTH Checklist Compliance Table

**Participant flow**	13a	Numbers randomly assigned, received treatment, analysed	No eHealth-specific additions	12–13	Figure 1 shows participant flow; 80 enrolled; attrition reported at each timepoint (Table 2, p. 21)
**Participant flow**	13b-i	Losses and exclusions after randomisation	Attrition diagram or usage/engagement figures	36–37	Engagement data reported: 42.5% did not access program; median 7/30 modules; 57.5% of users completed no topics
**Recruitment**	14a-i	Dates defining recruitment and follow-up periods	Indicate if critical secular events fell into study period	8	COVID-19 pandemic noted as significant secular event affecting trial design and interpretation
**Recruitment**	14b	Why the trial ended or was stopped	No eHealth-specific additions	–	Not applicable
**Baseline data**	15-i	Baseline demographic and clinical characteristics table	Report demographics associated with digital divide	13–14	Table 1 reports age, gender, education, employment, indigeneity, disability severity, K10 distress levels
**Numbers analysed**	16-i	Number of participants in each analysis	Report multiple denominators and definitions	20–21, 37	Table 2 provides sample sizes per measure at each timepoint; intent-to-treat N=80; engagement metrics reported
**Numbers analysed**	16-ii		Primary analysis should be intent-to-treat	27, 37	Primary analysis is intent-to-treat; all 80 enrolled participants included
**Outcomes & estimation**	17a-i	Results for each outcome with effect size and precision	Present process outcomes (use metrics and intensity)	31–34, 36–37	Tables 5–6 with estimates, SEs, 95% CIs; Figures 6–7; engagement data in secondary analyses
**Outcomes & estimation**	17b	For binary outcomes, absolute and relative effect sizes	No eHealth-specific additions	–	Not applicable – outcomes are continuous
**Ancillary analyses**	18-i	Subgroup and adjusted analyses	Subgroup analysis of users only	35–36	Covariate analyses with cognitive function; moderation by supporter and engagement; notes self-selection caveat
**Harms**	19-i	All important harms or unintended effects	Include privacy breaches, technical problems	41	Discusses fraudulent participation as a risk; notes challenges with online cognitive data collection
**Harms**	19-ii		Include qualitative feedback on strengths/shortcomings	–	Qualitative feedback was not sought (stated on p. 25)
**DISCUSSION**					
**Limitations**	20-i	Trial limitations, sources of potential bias	Typical limitations in eHealth trials	39–41	Discusses lack of blinding, single-arm design, selection bias, low engagement, measurement validity, online consent limitations
**Generalisability**	21-i	Generalisability of trial findings	Generalisability to other populations	39–40	Notes sample of motivated individuals likely reflects naturalistic uptake; discusses generalisability to general internet population
**Generalisability**	21-ii		Discuss elements in RCT different from routine application	15–16, 40	Discusses self-guided nature; no therapist involvement; low engagement suggests need for blended care in routine settings
**Interpretation**	22-i	Interpretation consistent with results	Restate study questions and summarise answers from data	37–39	Restates aims; summarises null findings on primary outcomes; discusses engagement and process outcomes

CONSORT-EHEALTH Checklist Compliance Table

**Interpretation**	22-ii		Highlight unanswered questions, suggest future research	38–43	Suggests gamification, social features, blended care, better fraud detection, improved cognitive assessment methods
**OTHER INFORMATION**					
**Registration**	23	Registration number and name of trial registry	No eHealth-specific additions	26	ANZCTR: ACTRN12620000113954
**Protocol**	24	Where full trial protocol can be accessed	No eHealth-specific additions	8	Published protocol referenced [27]
**Funding**	25	Sources of funding and other support	No eHealth-specific additions	44	NSW Dept of Health; Australian Commonwealth Dept of Health; Australian Rotary Health; Australian Foundation for Mental Health Research
**Competing interests**	X27-i	Declaration of interests	State relation of study team towards system being evaluated	44	Authors declare no conflicts of interest; authors are evaluators and developers (implied through affiliations)
